# Classifying protein-protein interaction articles using word and syntactic features

**DOI:** 10.1186/1471-2105-12-S8-S9

**Published:** 2011-10-03

**Authors:** Sun Kim, W John Wilbur

**Affiliations:** 1 National Center for Biotechnology Information, National Library of Medicine, National Institutes of Health, Bethesda, MD 20894, USA

## Abstract

**Background:**

Identifying protein-protein interactions (PPIs) from literature is an important step in mining the function of individual proteins as well as their biological network. Since it is known that PPIs have distinctive patterns in text, machine learning approaches have been successfully applied to mine these patterns. However, the complex nature of PPI description makes the extraction process difficult.

**Results:**

Our approach utilizes both word and syntactic features to effectively capture PPI patterns from biomedical literature. The proposed method automatically identifies gene names by a Priority Model, then extracts grammar relations using a dependency parser. A large margin classifier with Huber loss function learns from the extracted features, and unknown articles are predicted using this data-driven model. For the BioCreative III ACT evaluation, our official runs were ranked in top positions by obtaining maximum 89.15% accuracy, 61.42% F1 score, 0.55306 MCC score, and 67.98% AUC iP/R score.

**Conclusions:**

Even though problems still remain, utilizing syntactic information for article-level filtering helps improve PPI ranking performance. The proposed system is a revision of previously developed algorithms in our group for the ACT evaluation. Our approach is valuable in showing how to use grammatical relations for PPI article filtering, in particular, with a limited training corpus. While current performance is far from satisfactory as an annotation tool, it is already useful for a PPI article search engine since users are mainly focused on highly-ranked results.

## Background

The study of protein-protein interactions (PPIs) is one of the most critical issues in life-science research for understanding the function of individual proteins and the organization of biological processes. A plethora of biomedical literature that describes protein-protein interaction experiments by specifying individual interacting proteins and the corresponding interaction types exists. Since the vast majority of protein interaction information still exists in research articles, many efforts have been made to create protein interaction databases such as BIND [1], MINT [2], IntAct [3], and DIP [4]. However, several constraints such as the problems of manual curation of a database, the rapid growth of the biomedical literature, and of newly discovered proteins, make it difficult for database curators to keep up with the published information [5].

The BioCreative (Critical Assessment of Information Extraction Systems in Biology) challenge is a community-wide effort to build an evaluation framework for assessing text mining systems in biological domains [6]. PPI tasks were specially designed to study the detection of protein-protein interactions from literature, which have two subtasks in BioCreative III, ACT (Article Classification Task) and IMT (Interaction Method Task). ACT is the task to choose relevant abstracts to PPIs. IMT is the task to find experimental evidence of interacting protein pairs. Particularly, ACT is important since filtering PPI-relevant articles is a fundamental step for building annotation databases. Thus, high performance ACT systems can help reduce the curation burden at the initial curation stage.

Various approaches have been proposed to extract PPI information from biomedical literature. One popular method is to use predefined phrase patterns or to exploit co-occurrence of two protein names from text. These methods, however, have inherent limitations because they only find predefined PPI patterns, and are not able to discover new patterns. Machine learning (ML) techniques can discover new patterns not captured in a known trigger word list. Hence, ML approaches have gained popularity in recent years. Support vector machines (SVMs) have been widely used, and demonstrated outstanding performance [7-9]. Naive Bayes, k-nearest neighbor, decision trees, and neural networks have been alternatively used to extract PPI information [7,9]. Natural language processing (NLP) is a strategy utilizing linguistic features obtained from text, and also has been used for PPI extraction [10-14], where PPI sentences are assumed to have unique grammatical structures. However, the effectiveness of using parsing information has been little investigated at the article classification level.

Here, we present the method and the results from our participation in the BioCreative III ACT competition [15,16]. Our main focus on this task was to explore the effectiveness of applying word and grammatical features for our supervised learning approach to PPI article classification. It includes minimizing external knowledge other than training set such as templates or rule-based approaches developed on other tasks, and external databases, e.g., gene/protein dictionaries or full text information. The proposed method combines NLP strategies with ML techniques to utilize both word and syntactic features from text. To obtain gene names, articles are first tagged using a Priority Model [17]. This step is essential because protein names are the most important words triggering PPI descriptions. The gene-tagged articles are further analyzed to obtain word and syntactic features.

**Figure 1:**
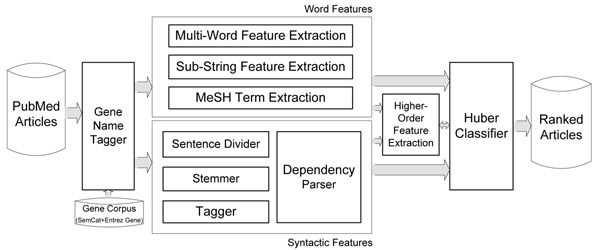
**Overview of the proposed PPI article classification approach.** Input articles are first evaluated whether there are gene/protein names in the text. After gene name detection, feature generation is performed in three different ways: word features including multi-words, sub-strings, and MeSH terms; syntactic features involving grammar relations between words; higher-order features obtained by evaluating a combination of different features.

For word features, multi-words, sub-strings, and MeSH (Medical Subject Headings) terms are applied for classifier input. Multi-word features are unigrams, bigrams, and trigrams of words. Sub-string features are sub-strings with *n* characters, which may help reduce the difference between distributions on training and test sets [18]. MeSH terms are also considered word features since MeSH is a controlled vocabulary for indexing and searching biomedical literature [19]. For syntactic features, the dependency relationships between words are mainly investigated. By using a dependency parser [20], a head word and a dependent word are determined as a two-word combination. This combination increases the problem space by increasing the total number of features. Therefore, we anonymize the gene names in dependent word positions by replacing with a special tag, e.g., ‘PTNWORD’. This process reduces the total number of features while leaving dependency information intact. Another aspect of features considered is to extract higher-order patterns by evaluating a set of feature combinations. When the proposed system predicts a part of the training corpus incorrectly, each feature combination is evaluated by a sum of partial derivatives of the loss function terms on data points [21]. This adds candidate features detected as potentially useful for the classification task. The last step is to learn article classification based on the extracted word and syntactic features. The constraint here was to minimize computational cost and processing time, but with reasonable classification performance. To achieve this purpose, a large margin classifier with Huber loss function [22] was adopted. Figure 1 depicts the overview of the proposed approach.

Although the current approach has much room for improvement, it produced the top-ranked performance among all submitted runs in the BioCreative III ACT task. As a result, we found that, in our system pipeline, syntactic patterns along with word features can effectively help distinguish between PPI and non-PPI articles. Note that the only external resource we used for the task was gene name data for the Priority Model, so the learning was solely limited to the given training corpus, which was a series of BioCreative datasets.

The paper is organized as follows. In the next section, we describe the results of our submission on the BioCreative ACT task. This is followed by discussion and conclusions drawn from our experience in BioCreative III. Lastly, our methods employed are explained.

## Results

Our goal for the ACT task is to develop a data-driven system with minimal external resources. To achieve this goal, choosing the right corpus is critical, whereas available benchmark sets are very limited. For this task, we collected gold standard sets from previous BioCreative competitions in addition to the BioCreative III corpus. The PPI article classification task has been a major topic since BioCreative II. Although the number of examples is still small, we assumed it was large enough to learn common positive and negative PPI patterns. Table 1 shows the corpus name and the number of positive and negative examples used for learning and testing. BioCreative II (6,172 abstracts), Biocreative II.5 (1,190 abstracts), and BioCreative III training data (2,280 abstracts) were used as the training corpus for all submitted runs. The BioCreative III development set was alternatively used to add more PPI information for training. The development set is the articles selected from the same pool as the BioCreative test set, hence it was also used to tune our system for official submission. The final candidate set for training consists of 5,820 positive and 7,822 negative examples. The test set includes 910 positive and 5,090 negative examples, which is more imbalanced compared to training data. This imbalance problem is discussed later in the Discussion section.

**Table 1:** The corpus information used in our experiments.

Corpus Name	Positive Examples	Negative Examples	Total Examples
BioCreative II	3874	2298	6172
BioCreative II.5	124	1066	1190
BioCreative III Training Set	1140	1140	2280
BioCreative III Development Set	682	3318	4000

Total Training Set	5820	7822	13642

BioCreative III Test Set	910	5090	6000

BioCreative II, BioCreative II.5, BioCreative III training, and development sets were used as the training corpus for the ACT competition. While the training corpus is balanced, the BioCreative III test set is an imbalanced set with the number of negative examples about six times higher than the number of positive examples. Hence, for the official submission, system parameters were tuned for the BioCreative III development set.

To assess the performance of submitted results, the BioCreative III competition relies on various performance measures, accuracy, specificity (true negative rate), sensitivity (recall), F1 score, MCC (Matthews’ correlation coefficient) score, and AUC iP/R (the area under the interpolated precision and recall curve). However, we discuss official runs based on F1 score, MCC score, and AUC iP/R. F1 score and MCC score evaluate the performance of binary classification, and do not account for ranked results. AUC iP/R, on the other hand, measures the quality of ranked results. Accuracy is commonly used to evaluate classification performance, which counts true positives and true negatives against the total number of predictions. But, in an unbalanced-class setting, accuracy does not successfully measure classification performance because if the number of true cases is strongly biased toward the negative class, e.g., accuracy is high simply by producing all negative predictions. The F1 score provides a more balanced evaluation by averaging precision and recall. The MCC score also fairly evaluates binary classification since it uses all four cases, TP (true positive), TN (true negative), FP (false positive), and FN (false negative). In particular, it is known to be more stable in the unbalanced class cases [23]. The F1 score and the MCC score are calculated as follows:(1)(2)

Where  and . Unlike F1 and MCC scores, AUC iP/R rather evaluates the performance of ranked results by considering precision rates for all recall points. For ranking systems or search engines, the performance at high ranks is more important than overall ranking, hence AUC iP/R is a good indicator of ranking-based performance. In Discussion, we instead use average precision for the ranking performance because it measures ranking performance in a more conservative way. Average precision is the average of the precisions at the ranks where relevant documents appear. It corresponds to the non-interpolated AUC P/R score. It is generally a lower value than AUC iP/R, but also emphasizes the higher ranks.

We submitted five runs for the ACT task, each using the same pipeline, but with different data and detailed feature sets (Table 2). For Run 1 and Run 2, unigrams and bigrams were used as multi-word features. Dependency relations were used in original form after anonymizing dependent genes/proteins to ‘PTNWORD’. The difference between Run 1 and Run 2 was use of the BioCreative III development set, which is also the difference between Run 3 and Run 4. For Run 3 and Run 4, word trigrams were added as features. To reduce complexity and also to make various forms into a single one, all words in dependency relations were stemmed using the Porter stemming algorithm [24]. The stemming increases the probability of matching the same relation in different word forms. In addition, feature selection was performed by removing features less frequent than four. This feature selection prevents escalating the number of features by ignoring the least frequent patterns, which might be insignificant for PPI classification. However, less frequent patterns may be very specific forms for describing PPIs. Therefore, removing such patterns may result in a performance decrease. Run 5 used the same strategy as Run 3, but utilized higher-order feature combinations as introduced in Background. For higher-order features, only binary combinations between features were evaluated to better fit the training corpus. The partial derivative threshold for this approach was empirically optimized for the BioCreative III development set. Our system was originally designed to give ranked results, rather than labels. However, the system output was binarized by using the sign of the Huber classifier output.

**Table 2:** The feature combinations used for submitted runs on the article classification task

	BC3 Dev Set	Multi-word			MeSH Term	Stemmed GRs	Feature Cut	Higher Order
		UNI	BI	TRI				
Run 1		X	X		X			
Run 2	X	X	X		X			
Run 3		X	X	X	X	X	X	
Run 4	X	X	X	X	X	X	X	
Run 5		X	X	X	X	X	X	X

The training data used in official submissions includes all examples of previous BioCreative PPI article tasks. However, the BioCreative III development set was selectively added for training in different runs. Unigrams (UNI), bigrams (BI), and trigrams (TRI) were used as multi-word features. MeSH feature is unigrams and bigrams from MeSH terms. For grammar relations (GRs), stemming was performed on Run 3 through Run 5. Feature cut was performed based on the frequency threshold four.

Table 3 presents the official performance scores of our submitted runs. Run 2 performed the best in terms of accuracy (89.15%) and MCC score (0.55306). Run 4 performed the best in F1 score (61.42%) and AUC iP/R (67.98%). Both Run 2 and Run 4 utilize the BioCreative development set as an additional training source, and it helped increase performance by about 2% overall. Applying higher-order consecutive words, i.e., trigrams, grammar relation stemming, and feature selection did increase F1 score and AUC iP/R, but the differences between Run 2 and Run 4 are insignificant. This indicates that the techniques applied to Run 4 were successful in reducing the number of features, while leaving the performance level unchanged. Run 1 and Run 3 obtained worse results than those using the BioCreative development set, however, the recall rates of both cases were increased. This is because the training data used in Run 2 and Run 4 has a much higher number of negative articles. About 13% more positive examples are added and the negative examples are increased by 74% compared to Run 1 and Run 3. In particular, adding more negative examples enables better prediction to respond to the imbalanced test set, but decreases recall. Note that Run 1 and Run 3 are still top-ranked results following Run 2 and Run 4 among total 49 submissions in the ACT task. This means that current PPI filtering pipeline effectively classifies PPI articles with or without feature variations.

**Table 3:** Official scores for the ACT competition.

	Run 1	Run 2	Run 3	Run 4	Run 5
TP	580	516	553	531	565
FP	417	257	376	288	398
FN	330	394	357	379	345
TN	4673	4833	4714	4802	4692

Accuracy	0.8755	**0.8915**	0.8778	0.8888	0.8762
Specificity	0.9181	**0.9495**	0.9261	0.9434	0.9218
Sensitivity	**0.6374**	0.5670	0.6077	0.5835	0.6209
F1 score	0.6083	0.6132	0.6014	**0.6142**	0.6033
MCC	0.53524	**0.55306**	0.52932	0.55054	0.53031
AUC iP/R	0.6591	0.6796	0.6589	**0.6798**	0.6537

TP, FP, FN, and TN are true positive, false positive, false negative, and true negative, respectively. MCC means Matthews’ correlation coefficient measure. AUC iP/R means the area under the interpolated precision and recall curve. F1 score and MCC evaluate the performance of binary classification. AUC iP/R evaluates system performance in terms of ranked results.

For the submitted runs, our intention for dealing with gene names was to handle each gene name as a single entity. So, gene names having multiple words are not separable during parsing and the result is more precise gene anonymization. However, we found afterwards that this was not applied for the official runs, i.e., gene names having multiple words were not treated as a unit. Table 4 shows the corrected performance for Run 2 and Run 4 by fixing this gene handling issue. Run 2’ and Run 4’ are newly obtained results for Run 2 and Run 4 respectively. For both cases, the number of true positives are increased, which results in higher F1, MCC, and AUC scores. Here, Run 4’ has the best performance among all runs by increasing those scores up to 1%.

**Table 4:** Performance results for corrected PPI classification on the ACT test set.

	Run 2	Run 2’	Run 4	Run 4’
TP	516	529	531	556
FP	257	271	288	311
FN	394	381	379	354
TN	4833	4819	4802	4779

Accuracy	**0.8915**	0.8913	0.8888	0.8892
Specificity	**0.9495**	0.9468	0.9434	0.9389
Sensitivity	0.5670	0.5813	0.5835	**0.6110**
F1 score	0.6132	0.6187	0.6142	**0.6258**
MCC	0.55306	0.55722	0.55054	**0.56100**
AUC iP/R	0.6796	0.6806	0.6798	**0.6834**

Run 2’ and Run 4’ are the corrected performance results for Run 2 and Run 4 respectively. For the official runs, gene names consisting of more than a single word were not treated as a single entity. Only this issue was fixed for Run 2’ and Run 4’.

Run 5 utilized binary feature combinations to capture higher-order relationships between features. The performance in Run 5 changed very little compared to Run 1 and Run 3, which proves to be an unsuccessful attempt, and it is not as we expected. For Run 5, we did not have time to analyze and optimize for the submission. According to our post-workshop experiments, classification performance is very sensitive to higher-order feature combinations, and difficult to optimize. For Run 5, we simply found a weight threshold which retained as many features as possible and yet increased performance for the BioCreative development set. That resulted in a total of 286,547 features. In the Discussion, we further investigate the effect of higher-order features.

Given the time available for the task, the submitted runs are obviously not fully optimized results. We believe further improvement is possible based on the ACT development set and also the recently released gold standard test set. But, we did not have sufficient time to investigate all the options for optimizing the current system with both datasets. Overtraining classification performance on the development set leads to an overfitting problem and decreased classification performance on the test set. So, our tuning for submitted runs was centered rather on different data and feature combinations, not fine tuning for parameters and heuristic knowledge. The performance produced by our system shows that the strategy of using both word and syntactic features in our classification framework is a good combination for the PPI article classification task.

## Discussion

### Article filtering with imbalanced classes

One main issue in the BioCreative III ACT competition is the imbalance problem between the number of positive and negative articles. Negative examples in the ACT development set are 82.95% of the whole development set. In the BioCreative test set, the ratio goes up to 84.83%. However, the training corpus gathered from previous BioCreative competitions is rather a balanced dataset. To overcome this problem, we tried several approaches. The popular method to solve the imbalance problem on training data is balancing the number of training examples by over- or under-sampling [25,26]. This sampling technique can be utilized for the imbalance problem on test data. For example, the training corpus can be reorganized by over-sampling non-PPI articles or under-sampling PPI articles. Another approach for addressing the imbalance issue is the careful selection of negative examples from unlabeled data as an additional training source. This method is similar to active learning [27]. Also, cost-sensitive learning [27] can be used along with an ensemble machine with multiple classifiers. Nevertheless, those attempts were not successful for the BioCreative ACT task.

The performance drop with an imbalanced test set compared to a balanced one can be easily explained. Assuming there is a prediction system performing at 90% precision for balanced data, 10% of positive predictions are false positive cases. If negative examples of the same kind are increased by a factor of six, false positive predictions are six times higher than in the former case. That results in a precision drop to 60% from 90%. This imbalance problem affects most of the performance scores except for accuracy. Accuracy can remain high because of dominant negative examples as explained in the Results section. In our system, the classification performance on training data exceeds 96% F1 score and 99% average precision. But this cannot ensure high performance on unbalanced test data.

### Utilizing word and syntactic feature types

Table 5 presents the effect of applying grammar relations using different classifiers in the BioCreative ACT task. The table shows the average precision rates for the BioCreative III development set when single words and their dependency relationships are used. ‘SW’ means using single words as features. ‘GR’ means word-relation features. All classifiers were trained using the BioCreative II, BioCreative II.5, and BioCreative III training data, and were optimized for giving the best scores on both training and development sets. ‘SVM’ is the support vector machine classifier with linear kernel. ‘Huber’ is the large margin classifier used in this paper.

**Table 5:** Average precision rates when adding grammar relations to single words.

Feature Set	Naïve Bayes	SVM	Huber
Single Words (SW)	0.6169	0.6600	0.6646
Grammar Relations (GR)	0.6281	0.6391	0.6417
SW + GR	**0.6538**	**0.6726**	**0.6771**

The best score is obtained by using both single words and grammar relations for all classifiers. The used training data was BioCreative II, BioCreative II.5, and BioCreative III training corpora. The performance was measured for the BioCreative development set.

As shown in the table, adding word-word relationships to single-word features boosts up the performance by 3.7% in naïve Bayes classifiers. For SVM and Huber classifiers, the improvement is less, however it shows that word dependency provides a positive effect for PPI article classification. The Huber classifier is the chosen approach for both data scalability and classification performance. Based on the performance comparison in Table 5, our Huber approach produces the best average precision overall.

**Figure 2:**
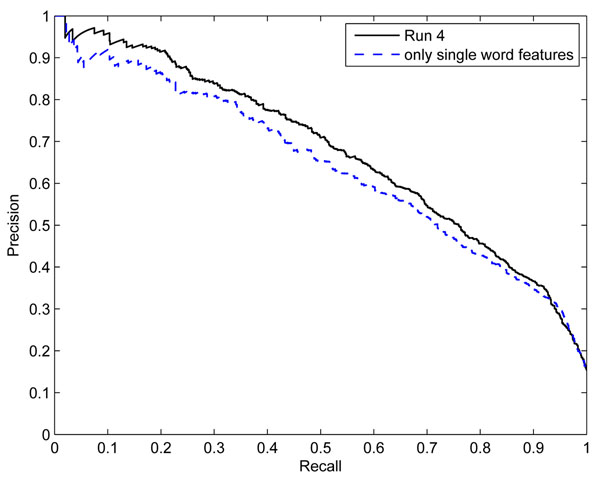
**The non-interpolated precision-recall curve on the BioCreative III test set.** The precision-recall curves show Run 4 and the result with single word features alone in the same classification pipeline. The points are the non-interpolated precision/recall value pairs obtained by the official BioCreative III evaluation script.

For the BioCreative ACT task, possible feature candidates were tested and analyzed including both word and syntactic features. As a result, five feature types were further selected for better classification. Table 6 shows the performance changes on the BioCreative III development set by varying those five feature types, gene anonymization, multi-words, sub-strings, MeSH terms, and higher-order features. The baseline performance is the result when Run 4 settings are applied. A row shows the evaluation results when all of the features without the feature type at that row are used. Since the higher-order features were not used in the Run 4, the features were rather added to the baseline in the last row of the table. We tried several feature combinations of the five feature types, but it was difficult to understand what feature type contributes more than others. Hence, the performance table was drawn from those simple variations. According to the table, removing each feature affects average precision and F1 score differently. For average precision, MeSH terms, gene anonymization, and sub-strings contribute positively, but for F1 score, gene anonymization contributes more. However, the feature contribution differs greatly depending on methods used and parameters. Figure 2 shows the non-interpolated precision-recall curve performance on the BioCreative III test set. The precision-recall curves present Run 4 and the result with single word features alone in the same classification pipeline. It is clearly seen that the word and syntactic feature types used in this paper improve the classification performance at most recall points.

**Table 6:** Performance changes on the ACT development set by varying feature types.

Used Features	Avg Prec	Precision	Recall	F1 score
Baseline	0.7073	0.6403	0.6290	0.6346
–Gene Anonymization	0.7017	0.6166	0.6320	0.6242
–Multi-words	0.7035	0.6358	0.6349	0.6354
–Sub-strings	0.7019	0.6329	0.6320	0.6324
–MeSH Terms	0.7009	**0.6410**	0.6334	0.6372

Baseline+Higher Order	**0.7077**	0.6311	**0.6496**	**0.6402**

The baseline performance is the result obtained from our system pipeline with the same setting used for Run 4. A row shows the evaluation results when a specific feature type is not used for the experiment. However, the last row is the performance results when higher-order features are applied.

The system reaches top performance on the BioCreative III development set when baseline and higher-order features are both used, which is the setting in Run 5. However, higher-order features are not easy to tune. More importantly, higher-order features do not provide the best result for the BioCreative III test set. In the proposed approach, gene name detection is a critical component of the system since gene names are handled individually and gene anonymization is based on this gene detection. During the BioCreative III period, we found some flaws of the Priority model in detecting correct gene names. Therefore, current performance is also limited by this detection capability.

### Ranking system for PPI article classification

In a binary classification system, F1 and MCC scores are useful to evaluate system performance. But, in a ranking system, top-ranking performance is more important than overall ranking. AUC iP/R and average precision are sensitive indicators for ranked results, and our system was basically tuned to achieve better average precision (AUC P/R) for submitted results. The best AUC iP/R score we obtained from official results is 0.6798, whereas the average AUC score of all participants is 0.4975 and the median AUC score is 0.5367. The precision-recall curves between our system and others also show significant differences in top-ranking results (http://www.biocreative.org/resources/biocreative-iii/workshop-talks). Figure 2 depicts the precision-recall curve for Run 4. The precision is over 90% until reaching 22% recall. Another perspective of ranking performance is the precision at rank *n* (*P*@*n*). For Run 5, *P*@100, *P*@200, and *P*@300 are 94%, 92%, and 85%, respectively. This shows that the proposed approach is effective for a ranking-based search system even though the overall performance is far from fully automating PPI article selection for annotation [15].

## Conclusions

In the paper, we present our system and its performance for the BioCreative III ACT competition. Our focus for the task was to develop a machine learning framework to effectively capture PPI articles from biomedical literature with minimal external resource use. The main idea here is detecting gene names and utilizing word-to-word relationships for automatically learning unique PPI patterns. The proposed approach identifies gene names by a Priority Model, and dependency relations are extracted by analyzing grammatical structures in sentences. A large margin classifier using the Huber loss function is used to learn from extracted word and syntactic features. Data scalability was also considered in selecting Huber classifiers for expanding target data to the whole PubMed corpus in the future.

Different feature types, including multi-words and grammar relations with stemming, and feature selection were exploited for submitted runs. Different training corpora were also used. Higher-order features were studied to see the possibility of automatic feature expansion. Through these studies, we found that syntactic features are useful at the article classification level as well as at the sentence classification level. Even though there is a limit to detection of correct gene names and the system is not optimized enough for the imbalanced nature of the dataset, the proposed system performs well in both binary classification performance and PPI ranking performance in all different data and feature combinations.

Current classification performance was achieved by only using a data-driven model containing different types of machine learning techniques. However, in the current setup, identifying gene names and analyzing dependency relationship are critical components, which need careful setup through utilizing PPI-related heuristic knowledge. Solving how many higher-order features may help for the PPI classification task is also a remaining issue. As a fully automatic annotation tool, the state-of-the-art systems are still far from real-world use. But, they can be utilized as support systems for manual curation. In particular, based on the BioCreative III ACT performance, our system is already useful for PPI article search in a Web environment.

## Methods

### Gene name detection using a Priority Model

In the proposed approach, gene names are identified using a Priority Model, which is a statistical language model for named entity recognition [17]. For named entities, a word to the right is more likely to be the word determining the nature of the entity than a word to the left in general. The Priority Model was constructed to follow this rule.

Let *T*_1_ be the set of training data for class *C*_1_ and *T*_2_ for class *C*_2_. Let {*t_α_*}*_α_*_∈_*_A_* denote the set of all tokens used in names contained in *T*_1_ ∪ *T*_2_. For each token *t_α_*, *α* ∈ *A*, it is assumed that there are associated two probabilities *p_α_* and *q_α_*, where *p_α_* is the probability that the appearance of the token *t_α_* in a name indicates that name belongs to class *C*_1_ and *q_α_* is the probability that *t_α_* is a more reliable indicator of the class of a name than any token to its left. Let *n* = *t*_α(1)_*t_α_*_(2)_⋯*t_α_*_(_*_k_*_)_ be composed of the tokens on the right in the given order. Then the probability of *n* belonging to class *C*_1_ can be computed as follows:(3)

To obtain *p_α_* and *q_α_*, a limited memory BFGS method [28] and a variable order Markov model [17] are used. For gene name detection, it is hard to get noise-free positive and negative names, however we used previously built data, SemCat [17] and Entrez Gene data, as an additional source to learn gene names.

There are common mistakes misclassified as gene names, e.g., mutant and protein, when this model is used. But, adding manual corrections might produce unexpected bias and it was not our intention for the ACT system. Thus, we only added a simple rule that a string with all numbers is not a gene name, which is one of misclassified cases by the learned model. Furthermore, only noun phrases were tested to minimize computation time and detection errors.

### Choosing word features

Feature generation is the most important part in machine learning problems. For PPI article classification, we emphasize the utilization of grammatical features in addition to our machine learning framework. However, individual words in text are always a good indicator to recognize PPI evidence. To investigate effective features for the ACT evaluation, we study three different types derived from the training corpus.

1. Multi-words: multi-word features are commonly known as *n*-grams. Since protein names sometimes contain more than a single word and since PPI is the interaction between proteins, *n*-consecutive words can be a good hint to divide PPI and non-PPI articles. Hence, we use the word combinations, unigram, bigram, and trigram. Only neighbor words are considered because long-distance word relationships are already estimated by syntactic features. Too many consecutive words also increase the problem space exponentially without performance improvement.

2. Sub-strings: while the basic elements of multi-word features are words, those of string features are characters, i.e., alphabetic and numeric. In biomedical literature, many entities appear in variant forms. Also, there is a report that the difference between distributions on training and test sets in PPI tasks can be reduced by considering character-based features [18]. Therefore, different character lengths from four to seven were tested for the ACT development set, and 6-consecutive characters produced slightly better results than other cases. For our submissions, six characters were used.

3. MeSH terms: the available training corpus is a set of PubMed articles, which have several fields for each record. The categories include journal title, article title, author list, abstract, MeSH, and article ID. Article title and abstract are the text we mainly used for word and syntactic feature extraction. MeSH terms are the additional source utilized for the PPI task. MeSH is a controlled vocabulary for indexing and searching biomedical literature [19]. MeSH terms are organized in a hierarchical structure and are used to indicate the topics of an article. Thus, this controlled vocabulary set can be helpful to find PPI-relevant articles.

**Figure 3:**
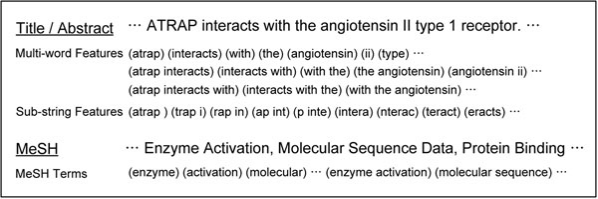
**An example of word feature extraction.** Unigrams, bigrams, and trigrams of words are selected as multi-word features. The sub-string feature contains six-consecutive characters. MeSH terms are extracted from the MeSH field in each article and unigram and bigram subphrases are used.

Figure 3 shows an example of how word features are constructed.

### Choosing syntactic features

Selecting PPI articles requires determining those articles describing physical protein-protein interactions. However, the direct relation between proteins is hard to determine considering the complex nature of sentences in PubMed documents and traditional word pattern matching has limits without semantic analysis [29,30]. To partially handle these problems without much effort in adding heuristic knowledge, we adopt a dependency parsing technique to extract the relationship between words along with gene name identification and anonymization. Using the word features such as multi-words and sub-strings helps recognize proximal word relations, whereas syntactic features based on grammar relations help discover long-distance word relationships as well as a more precise analysis at short-distances.

**Figure 4:**
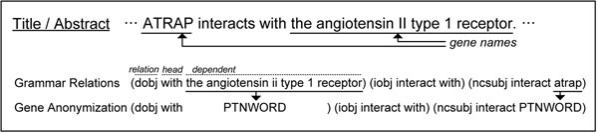
**An example of syntactic feature extraction.** Syntactic features are to analyze word-word relationships in a grammatical way. The words in each relation have different roles as a head word and a dependent word. To respond to general patterns, an anonymization technique is applied for gene names in the dependent position.

1. Dependency parsing: the C&C CCG parser [20] was used to obtain dependency relations. The software was publicly available and easy to attach to our library. Since we detect gene names beforehand, each gene or protein name can be handled individually. The output of the parser for a sentence is a set of dependency relations, which each contain a grammar relation name, a head word, and a dependent word. So, the head word is coupled with the dependent word by the specific relationship. However, extracted patterns are very sparse considering the size of the training corpus, hence we use an anonymization technique for gene names.

2. Gene name anonymization: the purpose of PPI article classification is to identify whether an article contains PPI information, not a gene or protein name itself. Therefore, in a dependency relationship, particular protein names are not so important. The gene name anonymization is a simple strategy to exchange a detected gene/protein word for a special tag, e.g., ‘PTNWORD’. This technique decreases the complexity of relationship features, while the relationship information remains the same. Figure 4 shows an example sentence and its syntactic features sets used in our approach.

### Choosing higher-order features from feature combinations

Even though manually constructed feature types are effective for classification, latent relationships among features may not be utilized by machine learning classifiers. The higher-order feature approach we used automatically extracts a set of feature combinations to obtain better classification performance. When system prediction is incorrect, each feature combination is evaluated by a sum of partial derivatives of the loss function terms on the misclassified data points [21], i.e.,

1. After testing on training data using a trained classifier, generate all bigrams by paring any two features from misclassified articles.

2. A sum of partial derivatives of the loss function over the respective data points is evaluated.

3. Bigrams occurring at least a times and with a partial derivative at least *b* in absolute value are selected.

Here, the loss function *h* is the modified Huber loss function [22] used by our classifier approach. We set a and *b* to 4 and 340, respectively, for the official Run 5. These parameters were empirically chosen to produce the best classification performance on the BioCreative III development set.

### Huber classifiers

The Huber classifier [22,31] used in the BioCreative task is a variant of support vector machines [32]. This method determines feature weights that minimize the modified Huber loss function [22], which is a function that replaces the hinge loss function commonly used in SVM learning.

Let *T* denote the size of the training set. Let the binary feature vector of the *i*th pair in the training set be denoted by *X_i_*. Let *y_i_* = 1 if the pair is annotated as positive and *y_i_* = –1 otherwise. Let *w* denote a vector of feature weights, of the same length as *X_i_*. Let *θ* denote a threshold parameter, and let *λ* denote a regularization parameter. Then the cost function is given by:(4)

where the function *h* is the modified Huber loss function defined as follows:(5)

The values of the parameters, *w* and *θ* minimizing *C* are determined using a gradient descent algorithm. The regularization parameter *λ* is computed from the training set as follows:(6)

where 〈|*x*|〉 is the average Euclidean norm of the feature vectors in the training set. For the ACT task, the parameter *λ*' was roughly tuned to maximize average precision rates for the BioCreative development set. Based on these experiments, it was finally set to 0.0005 for submitted runs.

## Competing interests

The authors declare that they have no competing interests.

## Authors’ contributions

SK and JW proposed the idea and SK carried out the computational experiments and analysis. JW supervised the project and revised this manuscript. All authors read and approved the final manuscript.
